# A Comparative Study of Retinal Function in Rabbits after Panretinal Selective Retina Therapy versus Conventional Panretinal Photocoagulation

**DOI:** 10.1155/2015/247259

**Published:** 2015-10-07

**Authors:** Young Gun Park, Seungbum Kang, Ralf Brinkmann, Young-Jung Roh

**Affiliations:** ^1^Department of Ophthalmology, Yeouido St. Mary's Hospital, College of Medicine, The Catholic University of Korea, No. 10, 63-ro Yeongdeungpo-gu, Seoul 07345, Republic of Korea; ^2^Medical Laser Center Lübeck GmbH, Lübeck, Germany

## Abstract

*Purpose.* This study evaluates functional changes in electroretinographic findings after selective retina therapy (SRT) compared to panretinal photocoagulation (PRP) in rabbits.* Methods.* The right eyes of 12 Chinchilla rabbits received 200 laser treatment spots. The right eyes of six rabbits received SRT (SRT group), whereas the other six animals were treated using PRP on the right eye (PRP group). The eyes were investigated using full-field ERG 1 hour and 3 weeks after treatment. Histologic exam to assess the tissue response of lasers was performed on 3 weeks.* Results.* No significant changes in the mean ROD or CR b-wave amplitudes of the SRT lesions were evident, compared to baseline, 1 h after laser treatment (*p* = 0.372 and 0.278, resp.). In addition, the OPs and 30 Hz flickers of the SRT lesions were not significantly altered (*p* = 0.17 and 0.243, resp.). At 3 weeks, similar results were found. Comparing the two groups, the ROD b-wave amplitude was reduced in the PRP and SRT groups to 60.04 ± 4.2% and 92.32 ± 6.43% of baseline (*p* < 0.001). Histologically, there was no visible photoreceptor alterations on week 3.* Conclusions.* SRT in rabbit eyes induced less functional loss than PRP in both rod-mediated retinal function and cone-mediated retinal function. In addition, SRT irradiated eyes had no functional loss compared to its control.

## 1. Introduction

Retinal photocoagulation is a major therapeutic method for various retinal and choroidal diseases. After irradiation, the laser energy is converted into heat in the retina, which leads to thermal damage of the RPE and photoreceptors. In addition, secondary damage in the neuroretina and choroid can occur [1]. The complications of laser photocoagulation such as visual field defects, loss of color vision, and lower night vision after panretinal treatment can be related to photoreceptor destruction [2–4]. Previous studies showed that laser photocoagulation induced destruction of the outer retina histologically and a decreased retinal function on electroretinogram [5, 6].

Selective retina therapy (SRT) was introduced as a new laser treatment for retinal diseases using the concept, which is associated with RPE degradation. The purpose of the irradiation is to selectively damage the RPE without affecting the surrounding tissue such as the neural retina, photoreceptors, and choroid. The treatment goal is to stimulate RPE cell migration and proliferation into the irradiated areas and to improve the metabolism at the diseased lesions [7]. SRT has already been performed in patients with various retinal diseases, such as drusen, due to age-related macular degeneration, diabetic macular edema, and central serous chorioretinopathy [8–10]. With the laser parameters (pulse duration: 1.7 *μ*s; repetition rate: 100 Hz; maximum number of pulses in a burst: 30; maximum adjustable pulse energy: 400 *μ*J) used, no bleeding or scotoma was observed, as confirmed by microperimetry, thus demonstrating no adverse effects to the choroid or photoreceptors, respectively [7, 11]. Considering the photoreceptor-sparing effect of SRT, it could be another treatment option for aforementioned retinal diseases.

SRT spots are invisible on ophthalmoscopy. However, these spots can be visualized by fluorescein angiography. So, dosimetry like reflectometry can be useful to prevent thermal damage to adjacent cells and to monitor the irradiated laser energy. This technique will allow an adequate treatment of retinal diseases for each individual with different pigmented fundus.

An optoacoustic (OA) device has been used as a dosimetry tool in previous SRT studies. The OA device is an instrument that detects pressure waves according to the collapse of microbubbles induced in the melanosome of RPE cells by laser irradiation. This device generally consists of an ultrasonic transducer embedded in an ophthalmologic contact lens. In contrast, reflectometry is a real-time controlled method for determining changes in the reflectance properties without embedding transducers. We previously reported the safety of SRT by using feedback controlled reflectometry in animal study, demonstrating that SRT of the rabbit eye induced selective RPE damage, as confirmed by both optical coherence tomography (OCT) and histological evaluation [12]. However, no detailed analysis of retinal function after SRT using real-time automated reflectometry in an experimental setting has been performed. In the present study, we investigated whether SRT preserved general retinal function compared to conventional panretinal photocoagulation (PRP). We used standardized protocols for full-field electroretinography (ERG) and histologic evaluation.

## 2. Materials and Methods

### 2.1. Animals

Twelve Chinchilla Bastard rabbits were used in this study, because pigmentations in RPEs in the retina are considerably similar to those in the human eye. We designed to use animals (4–6 months old, 2.0–2.5 kg) to have the similar levels of retinal maturation. The animals were used in accordance with the Association for Research in Vision and Ophthalmology (ARVO) statement for the use of animals in ophthalmic and vision research. The experiment was approved by the Institutional Animal Care and Use Committee of the Catholic University of Korea (Permit Number: YEO20131704FA). The animals were randomly assigned to two groups (both *n* = 6) and were treated using either an SRT laser (SRT group) or a PRP laser (PRP group). Only the right eyes were treated and the left eyes served as controls.

### 2.2. Laser Treatment

In the SRT group, the animals were treated using an SRT Laser (Q-switched Nd:YLF Laser: wavelength: 527 nm; pulse duration: 1.7 *μ*s; repetition rate: 100 Hz; maximum number of pulses in a burst: 30; maximum adjustable pulse energy: 40 *μ*J; number of spots: 200; laser spot diameter: 200 *μ*m). In contrast, eyes of the PRP group received conventional PRP by using Zeiss (VISULAS 532s, Carl Zeiss, Dublin, CA, USA; pulse duration: 200 ms; laser power: 100 mW; number of spots: 200; laser spot diameter: 200 *μ*m), inducing ophthalmoscopically visible lesions. All laser photocoagulation using contact lens (TransEquator; Volk Optical Inc., USA) was done below the optic nerve head along a horizontal marker to the medullary rays after the animals were sufficiently anesthetized by 0.2 mg/kg of Zoletil (250 mg of tiletamine and 250 mg of zolazepam; Virbac, Carros, France) administered intramuscularly.

### 2.3. Reflectometry

**Figure 1:**
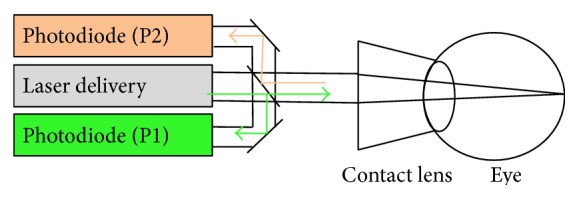
Schematic sketch of the reflectometry. The system contained two photodiodes; one of them (P1) detects the laser pulses applied to the fundus, and the other (P2) detects reflected and backscattered light from the retina. As a result, reflectometry can stop laser irradiation appropriately for selective RPE damage.

We used the same reflectometry system as previously described [12]. Briefly, reflectometry contained two photodiodes: one of them (P1) detects the laser pulses applied to the fundus and the other (P2) detects reflected and backscattered light from the retina. SRT emits 30 micropulses increasing stepwise by 3% of the dynamic range. However, reflectometry can stop laser irradiation when it detects microbubble formation in RPE. Given that microbubbles are the origin of instantaneous selective RPE damage, the backscattered light in the signal of P2 can be used as useful indicator (Figure 1). SRT provides that the pulse energy is increased stepwise from subthreshold intensity to a point of the selective RPE cell damage. For this purpose, the reflectometry system can adequately stop the laser emission as soon as microbubbles occur.

### 2.4. Electrophysiological Tests

The pupils of both eyes in each animal were dilated with topical 0.5% tropicamide and 0.5% phenylephrine hydrochloride (Mydrin-P ophthalmic solution; Santen Pharmaceutical Co., Ltd., Osaka, Japan). The animals were sufficiently anesthetized as aforementioned by 0.2 mg/kg of Zoletil (250 mg of tiletamine and 250 mg of zolazepam; Virbac, Carros, France) administered intramuscularly. The rabbits were dark-adapted for more than 1 hour, and a Burian-Allen bipolar contact lens electrode (Hansen Laboratories, Iowa City, Iowa, USA) was placed on the cornea. A needle ground electrode was inserted subcutaneously behind the ear.

Full-field electroretinograms were recorded and analyzed using the UTAS-E3000 system (LKC Technologies, Inc., Gaithersburg, MD), based on the protocols recommended by International Society for Clinical Electrophysiology of Vision. According to the protocols that have been described in detail previously [13], the dark-adapted rod responses were recorded with single flashes (20 *μ*s) on dim blue light background. On the other hand, combined rod and cone responses were measured using white light (0.8 cd s/m^2^). A 30 Hz flickering white light averaged from 20 sweeps was adequate to acquire light-adapted cone responses.

The amplitude of the b-wave measured from the trough of the a-wave to the following peak of the b-wave. And the implicit time measured from flash onset to the peak of the b-wave. Data are expressed as means ± standard deviation. Paired* t*-tests were performed to compare responses, and *p* values less than 0.05 indicated statistical significance.

### 2.5. Histologic Evaluation

On week 3, the animals were anesthetized as aforementioned and sacrificed by an overdose with KCL in the unconscious state to evaluate permanent changes of irradiated lesions rather than temporary changes. For immersion fixation in 4% glutaraldehyde, following the removal of the anterior parts of the eyes, the rest of the eye was prepared within 15 minutes immediately. The tissue was trimmed to block size after fixation for 12–24 hours in 4°C cold room. After dehydration in ethanol, the tissue was embedded. Sections with five micron thick were stained with hematoxylin and eosin. Histopathology was performed based on landmarks such as the optic nerve head and medullary rays to include the SRT lesions.

## 3. Results

### 3.1. Fundus Examination and Angiography

**Figure 2:**
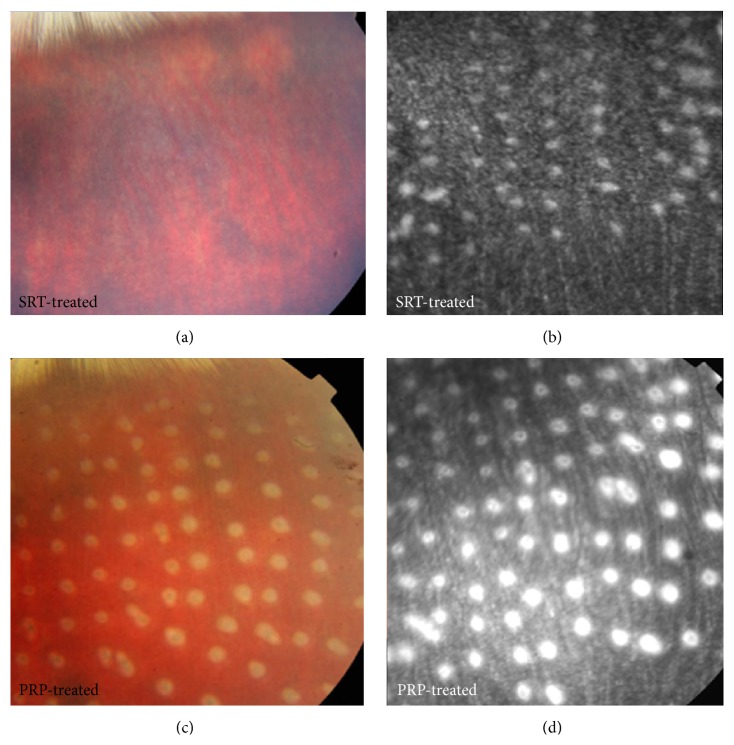
(a) A fundus image obtained 1 h after selective retina therapy (SRT) revealed no visible SRT spots. (b) Fluorescein angiography (FA) performed 1 hr after the irradiation showed hyperfluorescence on SRT spots. (c) A fundus photograph taken 1 hr after conventional PRP showed whitish spots. (d) FA performed 1 hr after PRP showed larger hyperfluorescence than that of SRT, although the same spot size (200 *μ*m) was applied.

In the SRT group, a total of 200 SRT laser spots were created in each rabbit (range, 10–40 *μ*J), and most of the test lesions were invisible. The representative fundus photographs obtained 1 hour after irradiation using SRT are shown in Figure 2(a).  Table 1 showed the energy levels of all lesions produced by SRT using reflectometric dosimetry. 1197 of total 1200 laser spots were evaluable.

Because these SRT lesions were invisible on ophthalmoscopy, SRT lesion can be detected by hyperfluorescence on fluorescein angiography (Figure 2(b)). In contrast, whitening lesions were immediately observed in the PRP group (range 100 mW) and were detected by fluorescein angiography. Although the same size of laser diameter (200 *μ*m) was used in both SRT and PRP groups, the lesion size of PRP is larger than that of SRT on FA (Figures 2(c) and 2(d)).

### 3.2. Electrophysiological Tests

The ERG recording results demonstrated general functional changes in the laser-induced retinal lesions after SRT and PRP. In the control groups, the mean ROD b-wave and combined response (CR) b-wave amplitudes were 106.3 ± 49.7 *μ*V and 157.6 ± 34.8 *μ*V, respectively. The mean oscillatory potential (OP) and 30 Hz flicker amplitude were 84.4 ± 23.4 *μ*V and 49.4 ± 17.8 *μ*V, respectively. At 1 hour and 3 weeks after SRT, the mean ROD b-wave amplitude of the SRT lesions was 98.2 ± 32.3 *μ*V and 101.2 ± 31.9 *μ*V, respectively, and the mean CR b-wave amplitude was 138.7 ± 29.4 *μ*V and 151.4 ± 23.0 *μ*V, respectively. The mean (OP) and 30 Hz flicker amplitude was 81.5 ± 31.4 *μ*V and 89.5 ± 42.5 *μ*V, respectively, at 1 h, and 47.6 ± 15.2 *μ*V and 46.3 ± 21.5 *μ*V, respectively, after 3 weeks (Table 2).

No significant changes in the mean ROD and CR b-wave amplitudes of the SRT lesions were evident 1 hour after laser treatment compared to baseline (*p* = 0.372 and 0.278, resp.). In addition, the OPs and 30 Hz flickers of the SRT lesions were not significantly altered 1 hour after SRT compared to baseline (*p* = 0.17 and 0.243, resp.). At 3 weeks, similar results were found for the ROD and CR b-wave amplitudes, OPs, and 30 Hz flickers (*p* = 0.142, 0.187, 0.435, and 0.272, resp.).

In contrast, at 1 hour and 3 weeks after conventional PRP, the mean ROD b-wave amplitude was 63.8 ± 33.5 *μ*V and 77.3 ± 22.8 *μ*V, respectively, and the CR b-wave amplitude was 109.5 ± 34.5 *μ*V and 111.3 ± 28.5 *μ*V, respectively. The mean OP and 30 Hz flicker amplitude at 1 hour were 48.6 ± 28.4 *μ*V and 43.5 ± 21.5 *μ*V, respectively, and on week 3 they were 26.8 ± 18.5 *μ*V and 14.4 ± 18.5 *μ*V, respectively. The ROD and CR b-wave amplitudes, OP, and 30 Hz flickers all significantly decreased 1 hour after conventional laser treatment compared to baseline (*p* < 0.0001, *p* < 0.0001, *p* = 0.023, and *p* < 0.0001, resp.). Similar results were also found on week 3 (*p* < 0.0001, *p* < 0.0001, *p* = 0.031, and *p* = 0.012, resp.). In the PRP group, apparent reductions in amplitudes were demonstrated 1 hour after SRT compared to baseline. These showed moderate recovery at 3 weeks but did not attain baseline values and remained significantly different.

Comparing the two groups, the ROD b-wave amplitudes in the PRP and SRT groups 1 h after irradiation were reduced to 60.0 ± 4.2% and 92.3 ± 6.4% of the baseline, respectively. This reduction was significantly greater in the PRP group compared to the SRT group (*p* < 0.001). The dark-adapted CR b-wave amplitude also showed similar results, which were a reduction in the PRP and SRT groups at 1 h (compared to baseline values) of 69.5 ± 5.4% and 88.0 ± 7.6%, respectively. The PRP group significantly decreased more than the SRT group (*p* < 0.05). OPs, cone single flash, and 30 Hz flicker responses were also significantly decreased in the PRP group, whereas the SRT group showed no statistically significant decrease. No statistically significant change in the ERG implicit time was observed after treatment in both groups.

**Figure 3:**
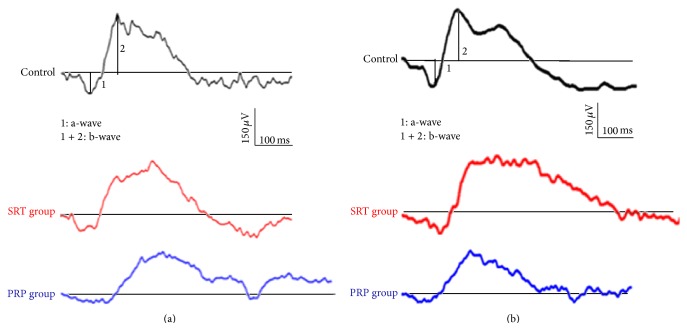
Dark-adapted ERG in the control group (top). ERG responses in the SRT groups were not significantly different from those of the control groups at either 1 h or 3 weeks after irradiation. (a, b, middle) However, the amplitudes of the conventional PRP group were significantly lower at these two time points (a, b, bottom).

**Figure 4:**
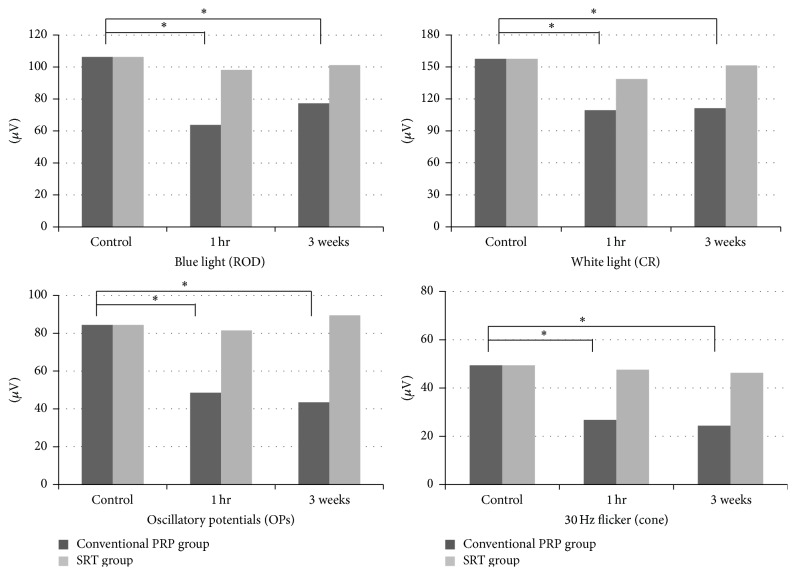
None of the b-wave amplitudes of the rods, CR, the amplitudes of the oscillatory potentials, or the 30-Hz flicker of the SRT group was significantly decreased. However, those of the conventional PRP group were significantly less than the control group.

At 3 weeks after treatment, similar results were observed. All parameters improved slightly compared to the values measured at the previous time point (1 h after irradiation), but the PRP group amplitudes were significantly reduced in comparison with those of the control group. Otherwise, no significant difference was noted between the SRT group and controls (Figures 3 and 4).

### 3.3. Histologic Evaluation

**Figure 5:**
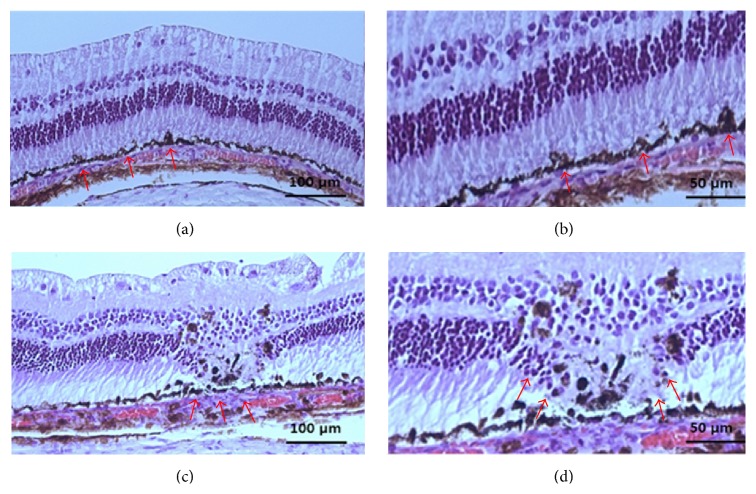
(a, b) The photoreceptor inner segments were preserved, and the distal ends of the photoreceptor outer segments were slightly relaxed. The RPE layer proliferated focally along the SRT lesion, and Bruch's membrane was intact. Histology of conventional laser lesions after 3 weeks. (c, d) The full-thickness structure of the retina involving the photoreceptors was disrupted and disorganized (magnification 200x; a, c) (magnification 400x; b, d) (red arrow, irradiated lesion).

In addition to the irradiation-outcome evaluation using ERG, a histologic examination was performed in order to access the extent of the laser lesions. At 3 weeks after irradiation, full-thickness retinal structures and arrangement were lost in the PRP group. In contrast, condensed focally proliferated RPE with preserved photoreceptors in the SRT lesions was observed. And the surrounding tissues such as Bruch's membrane and choriocapillaris were not affected by the SRT (Figure 5).

## 4. Discussion

Although conventional PRP is an effective treatment to reduce the risk of severe visual loss in Diabetic Retinopathy Study (DRS), it may cause visual acuity and peripheral visual field constriction due to neuroretinal damages [14, 15]. Moreover, conventional laser treatment develops irreversible damage on retinal tissue because the laser energy which is absorbed primarily by melanin in RPE conducts heat not only to RPE cells but also to the adjacent neurosensory retina.

SRT is a new laser technology that selectively damages the RPE while sparing neurosensory retinal tissue [16]. SRT was proved to reduce the risk of laser-induced scotoma induced by excessive thermal damage and has already been performed in patients with a range of retinal diseases [17–20]. In previous studies, preservation of the retinal anatomy was confirmed from histological findings and OCT images [12, 21]. However, the preservation of retinal function after SRT has not yet been evaluated, with the exception of one previous study that used microperimetry [11].

ERG is a useful examination method for study retinal function. The ERG b-wave is obtained primarily from the maximal combined response and reflects photoreceptor function. Physiologically, the b-wave results from the current flow along Muller cells in response to an increased extracellular potassium ion concentration. ERG is highly dependent on bipolar cells within the inner nuclear layer and hence on the retinal circulation. In the current study, we described retinal function using ERG and investigated whether SRT is associated with less retinal functional loss than PRP treatment.

In our study, the ERG of the SRT groups was not significantly changed at either 1 h or 3 weeks and generally recovered to baseline over 3 weeks. The subtle reduction in mean amplitudes measured 1 hr after SRT was not statistically significant. It was correlated well with the histopathological results showing no manifestation of retinal damage. This suggests that temporary RPE anatomical changes at the sites of SRT lesions become gradually resolved based on the histologic finding at 3 weeks. Histologically, we noted a focally proliferated RPE with relatively intact photoreceptors in the regions of irradiation at 3 weeks after irradiation. These findings support that SRT feedback controlled by reflectometry could induce RPE proliferation at irradiated lesions without photoreceptor damage as we published before [12]. In sections, Bruch's membrane and choriocapillaris were well preserved. The outer segments of the photoreceptors that were in contact with the RPE remained unaffected and it meant intact photoreceptor structure. In contrast, all of those of conventional PRP were significantly decreased at both time points, and these results also corresponded with the histologic findings. In conventional PRP lesions, full-thickness retinal layers were destroyed and disoriented. In addition, the decreased amplitude of OP and 30 Hz flickers at week 3 compared to 1 hour might reflect that both inner and outer retinal damages were intensified with permanent scarring process.

Anatomical changes in retinal tissue after SRT have been investigated in several previous studies [7, 12, 22, 23]. Selective RPE damage has been confirmed by histological findings and OCT images. To the best of our knowledge, this is the first electrophysiological study to compare SRT with conventional PRP using full-field ERG. The results of this study suggest that SRT using micropulsed duration and repeated pulses accompanied by real-time automated feedback dosimetry is fundamentally safe.

Roider et al. [24] found that multiple short laser pulses could selectively coagulate the RPE but with sparing of the adjacent neural retina and choroid. During the healing period, the epithelium reformed from a single sheet of hypertrophic retinal pigment epithelial cells that exhibited clear signs of viability, in that the cells phagocytized outer segments. The local edema of the photoreceptor layer and subretinal space evident at early stages disappeared when the blood-retinal barrier was reestablished and no subsequent damage to the photoreceptors was evident. In addition, many previous studies using SRT also reported temporary relaxation of the photoreceptor outer segment with subsequent restoration of the normal structure [12, 25, 26]. In the present study, preservation of the photoreceptor layer was noted in histological findings, which is in accordance with previous results. Therefore our work demonstrates that SRT did not significantly affect retinal tissue anatomy and function but rather induced reversible and temporary changes. These results suggest that SRT is a safe method for treating central macular disease, because the risk of laser-induced scotoma caused by irreversible thermal damage can be avoided and because any change to retinal function after SRT was reversible according to ERG.

Our study had several limitations. Full-field ERG cannot explore the functionality of each SRT spot but rather overall retinal functioning. Although multifocal ERG might be a better option for evaluation of individual SRT spots, the full-field ERG can be more suitable to examine an overall retinal function. Because of invisibility of SRT spot and the short distances between the spots, the spatial resolution would be far more below to see the specific laser-induced scotoma. Second, as the rabbit does not have a macula, our results are relevant to the effect of SRT on functioning of the overall retina, not the macula. Third, due to the tendency of laser effects which remain for a long period, a future study should be conducted to investigate the long-term effects of SRT.

In conclusion, SRT of microsecond duration featuring repeated pulses controlled by real-time automated reflectometry could preserve retinal function after laser irradiation compared to conventional PRP treatment, as verified by the ERG results. ERG can be an effective tool to evaluate the retinal function impairment and to comprehend the new treatment methods for the safety of SRT. In addition, retinal structures studied were not significantly damaged at 3 weeks after SRT. Furthermore, the changes in anatomy and function of retinal tissue after SRT were interrelated. The use of reflectometry as a dosimetry tool could avoid irreversible thermal damage during SRT by ensuring the appropriate energy delivery. Further studies are required to improve the safety of SRT by evaluating the functionalities of SRT lesions and to verify the efficacy of SRT applied to treat patients with various macular diseases.

## Figures and Tables

**Table 1:** The results of automatic turn-off in the selective retina therapy groups.

Energy of SRT^a^ (0–40 *μ*J)	Number of SRT^a^ lesions	Fundoscopically visible lesions (%)	FA^b^-positive lesions	Automatic turn-off
0–19	515	2 (0.39%)	515	510
20–39	674	5 (0.74%)	674	669
40	8	0	8	8
Total	1197	7 (0.58%)	1197	1190

^a^SRT, selective retina therapy; ^b^FA, fluorescein angiography.

**Table 2:** Full-field electroretinography (ERG).

Parameter	Control	PRP groups (*n* = 6)				SRT groups (*n* = 6)			
		1 h	*p* value	3 weeks	*p* value	1 h	*p* value	3 weeks	*p* value
Blue light (rod)	106.3 ± 49.7	63.8 ± 33.5	<0.0001^*∗*^	77.3 ± 22.8	<0.0001^*∗*^	98.2 ± 32.3	0.372	101.2 ± 31.9	0.142
White light (CR)	157.6 ± 34.8	109.5 ± 34.5	<0.0001^*∗*^	111.3 ± 28.5	<0.0001^*∗*^	138.7 ± 29.4	0.278	151.4 ± 23.0	0.187
Oscillatory potential	84.4 ± 23.4	48.6 ± 28.4	0.023^*∗*^	43.5 ± 21.5	0.031^*∗*^	81.5 ± 31.4	0.170	89.5 ± 42.5	0.435
30 Hz flicker (cone)	49.4 ± 17.8	26.8 ± 18.5	<0.0001^*∗*^	24.4 ± 18.5	0.012^*∗*^	47.6 ± 15.2	0.243	46.3 ± 21.5	0.272

^*∗*^Averaged values ± two standard deviations of the b-wave amplitudes (*μ*V). The two-tailed paired *t*-test was used to calculate *p* value, and ^*∗*^ *p* < 0.05 was considered to mean the significance.
